# Specific drugs for rare diseases in a province of eastern China under catalog management: from 2021 to 2023

**DOI:** 10.3389/fphar.2025.1476910

**Published:** 2025-03-31

**Authors:** Ruifang Nie, Zhen Zhao, Yahui Zhang, Bo Xu, Wen Zhang

**Affiliations:** ^1^ Department of Pharmacy, Shandong Provincial Hospital Affiliated to Shandong First Medical University, Jinan, China; ^2^ Finance Department, Shandong Provincial Public Resources Trading Center, Jinan, China

**Keywords:** rare diseases, China’s list of rare disease, catalog management, specific therapeutic drug, drug procurement

## Abstract

**Background:**

China attaches great importance to the prevention and treatment of rare diseases. The government has successively formulated two rare disease catalogs, and approved a variety of rare diseases treatment drugs. However, the actual supply and utilization of these drugs post-marketing remains unclear.

**Methods:**

Based on the first and second list of national rare disease catalog in China, this study sort out the specific therapeutic drugs and extract procurement data from the provincial platform over the past 3 years. Subsequently, the drug allocation, shortages, delivery rate, temporal changes, and spatial distribution were analyzed to gain a comprehensive understanding of the local drug supply situation.

**Results and Discussion:**

In the first catalog of 121 rare diseases, China has listed 54 specific drugs; in the second catalog of 86 rare diseases, 35 specific therapeutic drugs have been identified. Among these drugs, Shandong Province has access to 42 and 28, respectively. Spesolimab, Sodium Phenylbutyrate, Nitisinone and Emapalumab are currently in short supply, and the delivery rate of 16 drugs such as Selumetinib, Sirolimus (tablet), Octreotide, Dimethyl Fumarate and Lanreotide is below 80%. The number of available drugs increased year by year. The allocation of 19 drugs increased significantly, and 19 drugs were newly developed. The overall procurement cost of drugs increased and then decreased, which may be related to national policies. Additionally, there are significant regional disparities in drug cost, with Jinan, the provincial capital, leading at 770 million RMB.

**Conclusion:**

The number of specific drugs for rare diseases has steadily increased, with the drug availability rate in Shandong Province reaching 80%. This indicates a generally high level of accessibility to drugs for rare diseases in China. However, attention should be given to improving the supply capacity for drugs that are in short supply and have a low delivery rate.

## 1 Introduction

Rare diseases are a general term for a large class of diseases scattered in various disease systems, characterized by their low incidence and uncommon nature (The Lancet, 2020; The Lancet Neurology, 2022). These diseases are typically chronic and serious, often posing a significant threat to life (Chung et al., 2022; Rare diseases, 2022). Rare diseases are defined differently across countries (Richter et al., 2015; Adachi et al., 2023). Currently, the most widely adopted international approach for defining rare diseases involves establishing a threshold based on incidence rates or the number of cases. Diseases occurring below this threshold are classified as “rare” (Nguengang et al., 2020). In China, the current criteria specify an incidence rate of less than 1/10,000 among newborns, a prevalence rate of less than 1/10,000, or an affected population of less than 140,000 (Lu and Han, 2022).

Rare diseases have increasingly been recognized as a public health priority in China. On 11 May 2018, the National Health Commission, Ministry of Science and Technology, Ministry of Industry and Information Technology, National Medical Products Administration, and National Administration of Traditional Chinese Medicine jointly published China’s First List of Rare Diseases, which included 121 rare diseases (He et al., 2018). Subsequently on 18 September 2023, these five departments, along with the Logistics Department of the Central Military Commission, released the Second List of Rare Diseases, adding 86 diseases (Tang et al., 2023). Thus far, a total of 207 rare diseases have been included in the catalog management in China. The diseases listed in the catalog receive priority support from national medical insurance, local security initiatives, and incentives for drug research and development (Liu M. et al., 2024; Zhao et al., 2023).

Rare disease drugs are used for the diagnosis, prevention and treatment of rare diseases (Tambuyzer et al., 2020; Chen et al., 2024; Barrett et al., 2023). Due to the vast number of rare diseases, the smaller patient populations, limited market demand, and high research and development costs, fewer pharmaceutical companies focus on developing drugs for rare disease, leading to they are also called orphan drugs (Puig-Junoy and Campillo-Artero, 2019; Zhang et al., 2019). Multiple guidelines and consensuses have provided valuable insights into standardizing treatments for rare diseases (Burgos et al., 2024; Chen et al., 2021; de Graeff et al., 2019). Nevertheless, there remains a lack of clear policy regarding orphan drug qualification criteria as well as a mature list. Most existing studies focus on the marketing of orphan drugs, their efficacy, and comparative analyses between different regions (Giannuzzi et al., 2017; Gorini et al., 2022; Heard et al., 2020; Yang et al., 2019; Zhi et al., 2023; Liu et al., 2023; Liu Qingyang et al., 2024), but data on downstream drug supply and utilization are lacking. In particular, once relatively common drugs are excluded, the allocation of specific drugs can better reflect the effectiveness of local orphan drug supply systems. Therefore, this study aims to review the specific therapeutic drugs listed in the first and second rare disease catalog in China and analyze actual purchasing data from one eastern province. Our findings will provide a deeper understanding of local allocation and use of rare disease drugs, and offer insights for improving the capacity for treatment of rare diseases.

## 2 Materials and methods

### 2.1 Study subjects

Using the names of 121 rare diseases from the First List of Rare Diseases and 86 rare diseases from the Second List as keywords, we retrieved relevant diagnosis and treatment guidelines, expert consensus and references. We then summarized the therapeutic drugs accordingly. Drugs that are relatively common in clinical practice and primarily used for non-rare diseases were excluded. Examples of such exclusions include Hydrocortisone for treating 21-Hydroxylase Deficiency and Congenital Adrenal Hyperplasia, Lamotrigine for severe Myoclonic Epilepsy (Dravet syndrome) in infants, and Pemetrexed Disodium for Malignant Pleural Mesothelioma. Finally, a list of specific therapeutic drugs for rare diseases was compiled.

This process was conducted independently by two researchers to reduce the bias caused by personnel.

### 2.2 Data sources

The databases used in this study included PubMed, EMbase, China National Knowledge Infrastructure (CNKI), and Wanfang. The specific therapeutic drugs identified were cross-referenced and supplemented with information from drug labels, the 2019 Edition of the Rare Disease Diagnosis and Treatment Guide, the Rare Disease Information Network, and the official account of the China Rare Disease Alliance.

The specific therapeutic drugs described in Section 2.1 were searched through Shandong Provincial Public Resources Trading Center and Shandong Province Medical Security Bureau. Extraction and record China approved drug names, trade name, drug specifications, dosage forms, pharmaceutical packaging specifications, unit price, transaction price, name of medical institutions, medical institutions level, order number, order delivery quantity, actual delivery quantity, actual delivery time and delivery cities, production enterprises, distribution and other relevant information. The data collection period spans from 1 January 2021 to 31 December 2023.

### 2.3 Statistical analysis

The overall situation, shortages, temporal changes, and spatial distribution of specific therapeutic drugs for rare diseases were statistically analyzed using Excel software. For drugs available with multiple specifications and dosage forms, data were consolidated into the specifications with the highest purchase volume. Epidemiological data were obtained from Shandong Rare Diseases Registry System (SRDRS) or literature. The statistical methods in this study are defined by the following formulas:

Provincial drug coverage rate = number of specific therapeutic drugs available in the province/total number of specific therapeutic drugs listed in China.

Provincial disease coverage rate = number of diseases with specific therapeutic drugs available in the province/total number of diseases with specific therapeutic drugs listed in China.

Average Annual Growth Rate = (Number of drugs in 2023/Number of drugs in 2021) ^ (1/2) - 1.

Drug delivery rate = quantity requested/quantity actually delivered.

## 3 Results

### 3.1 Allocation of specific drugs

By analyzing the first and second lists of rare diseases, we identified the specific therapeutic drugs. The overall findings are summarized in Table 1.

**TABLE 1:** Specific drugs for rare diseases in China and in Shandong Province.

Catalog	Total diseases	China approved		Shandong allocation		Coverage	
		Disease	Drug	Disease	Drug	Disease	Drug
First	121	33	54	29	42	87.9%	77.8%
Second	86	26	35	20	28	76.9%	80.0%

Of the 121 diseases in the First List of Rare Diseases, 33 have specific therapeutic drugs approved in China, among a total of 54 drugs. Of these, 42 had procurement records in Shandong Province, covering 29 rare diseases (Supplementary Table S1). This indicates that Shandong Province has been equipped with 77.8% of the specific therapeutic drugs for the First List of rare diseases approved in China.

Of the 86 diseases listed in the Second List of Rare Diseases, 26 have specific therapeutic drugs approved in China, totaling 35 drugs. Among them, 28 drugs have procurement records in Shandong Province, covering 20 rare diseases (Supplementary Table S2). Consequently, Shandong Province has been equipped with 80.0% of the specific therapeutic drugs for the second list of rare diseases approved in China.

### 3.2 Shortages of specific drugs

According to Table 1, approximately 80% of rare diseases can be treated with specific therapeutic drugs in Shandong Province. However, an analysis combining with epidemiological information revealed that some rare diseases still lack specific drugs (Table 2). From July 2015 to 2022, 2 cases of N-acetylglutamate Syndrome Deficiency and 26 cases of Ornithine Carbamoyl Enzyme Deficiency were reported to SRDRS in Shandong Province. Unfortunately, these patients did not have access to the specific therapeutic drug, Sodium Phenylbutyrate locally. Additionally, 5 cases of Tyrosinemia and 2 cases of Familial Hemophagocytic Lymphohistiocytosis were reported in Shandong Province without procurement records for Nitisinone and Emapalumab. Furthermore, given the incidence rate of Generalized Pustular Psoriasis in China (1.403/100,000) and Shandong Province’s resident population of 101,627,900 in 2022,there could be an estimated 1,425 patients with this disease in Shandong Province (Feng et al., 2023). However, there are no purchase records for the therapeutic drug Spesolimab. Currently, there are no epidemiological reports on Malignant hyperthermia, Narcolepsy, Neurotrophic keratitis or Persistent pulmonary hypertension of the newborn in China, making it challenging to assess the demand for related drugs in Shandong Province.

**TABLE 2:** Shortage of specific drugs for rare diseases in Shandong Province.

Catalog	Entry	Rare disease	Drug	Patients^a^
First	79	N-acetylglutamate Synthase Deficiency	Sodium Phenylbutyrate	2^b^
	85	Ornithine Transcarbamylase Deficiency	Sodium Phenylbutyrate	26^b^
	115	Tyrosinemia	Nitisinone	5^b^
Second	26	Familial hemophagocytic lymphohistiocytosis	Emapalumab	2^b^
	33	Generalized Pustular Psoriasis	Spesolimab	1425^c^
	46	Malignant hyperthermia	Dantrolene Sodium	—
	52	Narcolepsy	Pitolisant	—
	56	Neurotrophic Keratitis	Cenegermin	—
	59	Persistent Pulmonary Hypertension of the Newborn	Nitric Oxide for Inhalation	—

^a^ Approximate number of patients.

^b^ Data from SNRDRS, date: 2015-2022.7.

^c^ Data from literature.

### 3.3 Drug delivery rate

**FIGURE 1:**
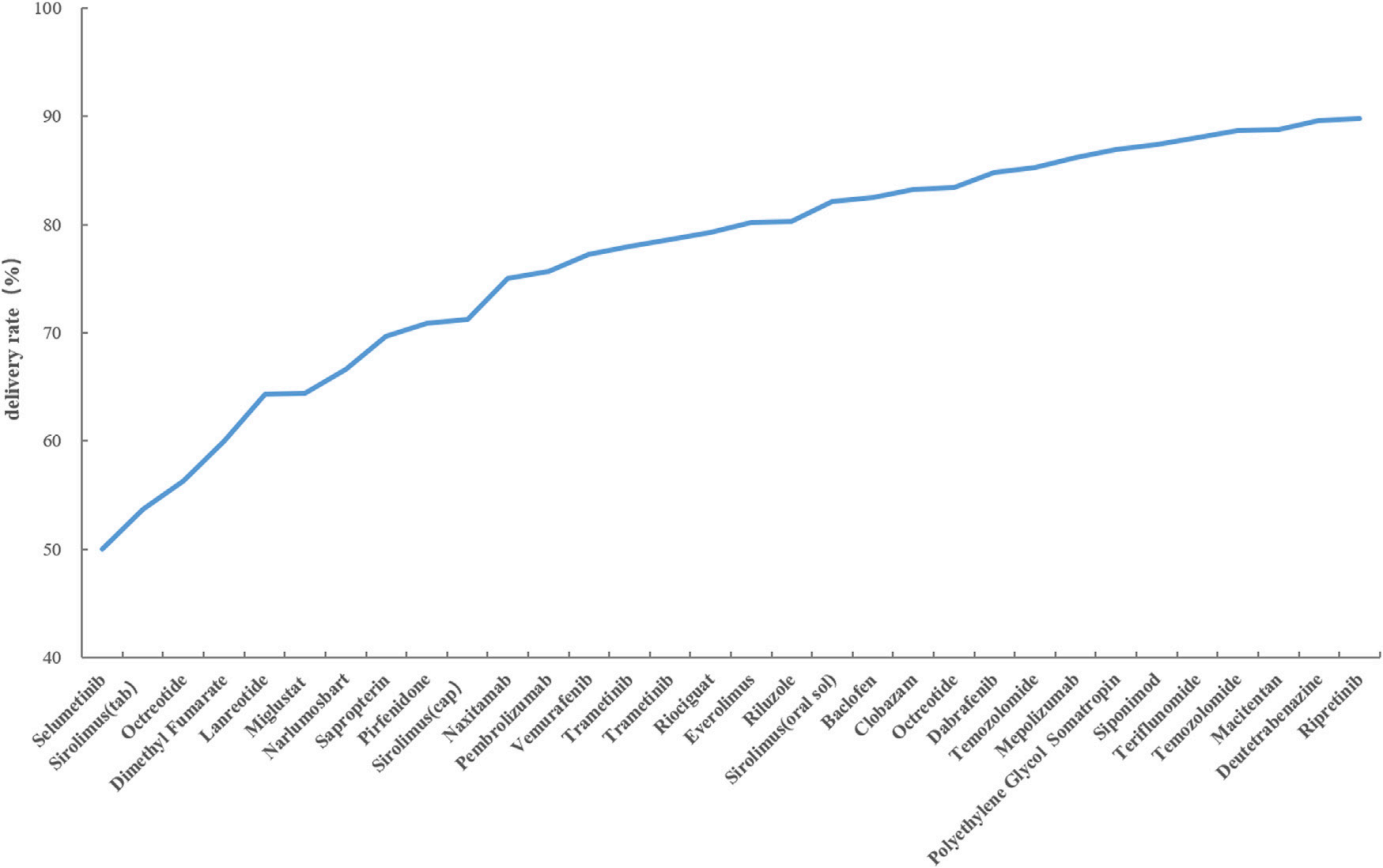
Delivery rate of rare disease specific drugs in Shandong Province.

Calculation of the drug delivery rate serves as an indicator of the extent to which drug demand is satisfied, thereby highlighting potential issues in the drug distribution process. This study illustrates drugs with delivery rates below 90% in Figure 1. The calculation of the drug delivery rate can reflect the degree to which drug demand is met, indicating potential issues in the drug distribution process. This study illustrates drugs with a delivery rate below 90% in Figure 1. It is evident that Selumetinib, Sirolimus (tablet), and Octreotide have delivery rates below 60%. Dimethyl Fumarate, Lanreotide, Miglustat, Narlumosbat, and Sapropterin fall within the range of 60%–70% for their delivery rates. Pirfenidone, Sirolimus (capsule), Naxitamab, Pembrolizumab, Vemurafenib Trametinib and Riociguat exhibit delivery rates between 70% and 80%. The remaining drugs have a delivery rate exceeding 80%.

### 3.4 Temporal changes

#### 3.4.1 General information

The number of specific drugs for rare diseases in Shandong Province has increased steadily over the years, rising from 44 in 2021 to 55 in 2022, and reaching 62 in 2023 (Table 3). Surprisingly, the overall cost has not shown a continuous increase. In fact, the total procurement in 2023 was 697.1 million RMB, slightly lower than the 795.8 million RMB in 2022. Apart from adjustment of the drug utilization structure, the reduction in drug unit price may be one of the significant contributing factors. For instance, under national policy, the price of Nusinersen Sodium Injection was reduced from 699,700 RMB to 33,180 RMB, and the price of Temozolomide capsules fell from 1,967.58 yuan to 246.5 RMB.

**TABLE 3:** Drug number and cost in Shandong Province.

Year	Drug number	Cost (/10^7^yuan)
2021	44	63.10
2022	55	79.58
2023	62	69.71

#### 3.4.2 Annual growth

A statistical analysis of the annual distribution of all drugs revealed that 19 drugs exhibited an average annual growth rate of over 50% (Table 4). Notably, Alirocumab injection for the treatment of Homozygous Hypercholesterolemia demonstrated the highest growth rate, surging from 103 in 2021 to 97,544 in 2023, with an average annual growth rate of 29.77 (Table 4, Entry 1). Meanwhile, the Evolocumab injection, another therapy, experienced an increase from 5,705 units in 2021 to 136,214 units in 2023, yielding an average annual growth rate of 3.89 (Table 4, Entry 5). For the treatment of Idiopathic Pulmonary Arterial Hypertension, the average annual growth rates for Treprostinil (4.40, Table 4, Entry 2), Riociguat (1.94, Table 4, Entry 9), and Selexipag (0.56, Table 4, Entry 17) indicate varying levels of market expansion. Additionally, the number of Nusinersen Sodium used for treating Spinal Muscular Atrophy rose dramatically from 18 in 2021 to 480 in 2023, demonstrating an average annual growth rate of 4.16 (Table 4, Entry 4).

**TABLE 4:** Drugs with significant growth in Shandong Province.

Entry	Drug	Specification	2021	2022	2023	Average annual growth
1	Alirocumab	1.0 mL:75 mg	103	33,347	97,544	29.77
2	Treprosti	20 mL:20 mg	5	2	146	4.40
3	Dinutuximab beta	20 mg (4.5 mL)	20	539	551	4.25
4	Nusinersen Sodium	5 mL:12 mg	18	804	480	4.16
5	Evolocumab	1 mL:140 mg	5,705	69,338	136,214	3.89
6	Ripretinib	50 mg*30	6	77	120	3.47
7	Agalsidase Alfa	3.5 mg (3.5 mL)	94	483	1762	3.33
8	Nintedanib	150 mg*30	1,008	3,671	14,253.67	2.76
9	Riociguat	1 mg*42	73	569	630	1.94
10	Alglucosidase Alfa	50 mg	1,155	3,341	5,370	1.16
11	Dabrafenib	75 mg*120	50	94	231	1.15
12	Denosumab	120 mg (1.7 mL)	14,085	45,352	59,563.5	1.06
13	Trametinib	2 mg*30	53	105	207	0.98
14	Brentuximab Vedotin	50 mg	54	120	186	0.86
15	Polyethylene Glycol Recombinant Human Somatropin	54 IU/9.0 mg/1.0 mL	732	1750	2,488	0.84
16	Teriflunomide	14 mg*28	21	7	68	0.80
17	Selexipag	0.2 mg*60	221	573	538	0.56
18	Melphalan	50 mg	184	288	447	0.56
19	Fingolimod	0.5 mg*28	6	13	14	0.53

#### 3.4.3 Newly available drugs

19 drugs were newly developed during the study period (Table 5). In 2023, Eculizumab Injection, a significant drug for the treatment of rare diseases with indications for Atypical Hemolytic Uremic Syndrome, Generalized Myasthenia Gravis, Neuromyelitis Optica, and Paroxysmal Nocturnal Hemoglobinuria, was launched in Shandong Province (Table 5, Entry 2). Furthermore, the availability of Mepolizumab (Table 5, Entry 3), Vigabatrin (Table 5, Entry 6), Selumetinib (Table 5, Entry7), Risdiplam (Table 5, Entry 9), Tafamidis (Table 5,Entry 12),and Clobazam (Table 5,Entry 19) has addressed the lack of corresponding treatments for rare diseases locally. Notably, Clobazam, Risdiplam, and Luspatercept have demonstrated significant growth.

**TABLE 5:** Newly available drugs in Shandong Province.

Entry	Drug	Specification	2021	2022	2023
1	Luspatercept	25 mg	0	0	246
2	Eculizumab	300 mg/30 mL	0	0	98
3	Mepolizumab	100 mg (1 mL)	0	0	94
4	Inebilizumab	100 mg (10 mL)	0	0	51
5	Naxitamab	40 mg (10 mL)	0	0	18
6	Vigabatrin	500 mg*30	0	0	5
7	Selumetinib	10 mg*60	0	0	2
8	Narlumosbart	120 mg (1.6 mL)	0	0	2
9	Risdiplam	60 mg	0	2	543
10	Ofatumumab	20 mg (0.4 mL)	0	3	54
11	Lanreotide	120 mg	0	7	11
12	Tafamidis	61 mg*30	0	10	69
13	Dimethyl Fumarate	240 mg*56	0	10	3
14	Fampridine	10 mg*56	0	11	2
15	lcatibant	3 mL:30 mg	0	15	16
16	Ipilimumab	50 mg (10 mL)	0	27	20
17	Siltuximab	100 mg	0	28	41
18	Idursulfase beta	6 mg (3 mL)	0	34	20
19	Clobazam	10 mg*28	0	45	2,623

### 3.5 Regional distribution

**FIGURE 2:**
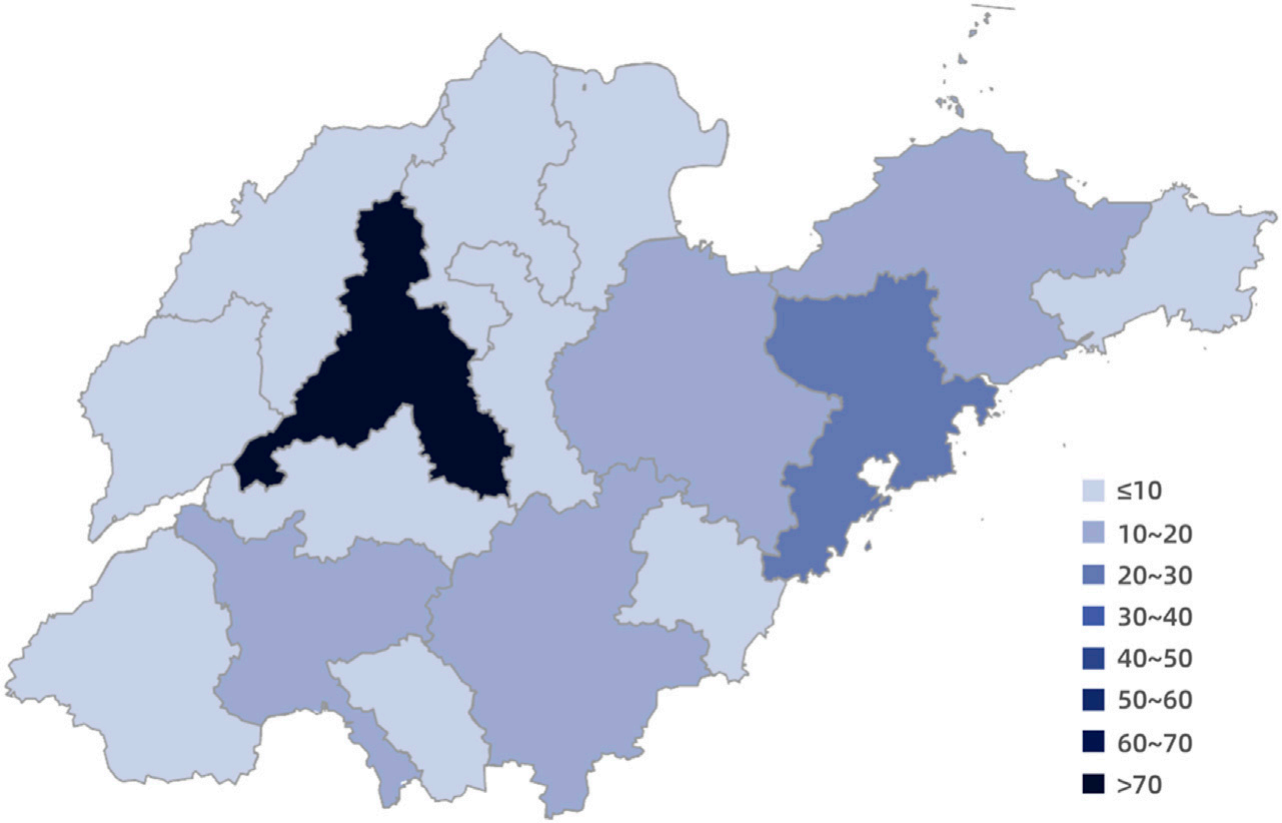
Regional distribution of the purchase amounts of specific drugs in Shandong Province.

Shandong Province, situated on the eastern coast of China, comprises 16 prefecture-level cities: Jinan, Qingdao, Zibo, Zaozhuang, Dongying, Yantai, Weifang, Jining, Taian, Weihai, Rizhao, Linyi, Dezhou, Liaocheng, Binzhou and Heze. An analysis of the procurement amounts for specific drugs used in the treatment of rare diseases across these 16 cities (Figure 2) revealed that the largest expenditure was in the provincial capital, Jinan, amounting to77*10^7^ RMB. The second highest procurement amount was found in Qingdao, totaling 22*10^7^ RMB, followed by Weifang, Linyi, Jining, and Yantai. In contrast, Weihai reported the smallest procurement amount, at just 3.26*10^7^ RMB.

## 4 Discussion

Since 2018, China has implemented a catalog management system for rare diseases, publishing two lists covering a total of 207 rare diseases. Similarly, Japan and South Korea have also adopted catalog-based management for rare diseases, confirming 338 and 1,147 rare diseases respectively (Feng et al., 2021; Yang et al., 2022). However, it is important to note that these catalogs cover only a fraction of the over 7,000 known rare diseases globally (Haendel et al., 2020). The groups of rare disease patients not included in these directories also warrant attention.

There have been several reports on the accessibility of orphan drugs in China, which indicate that the diagnostic level of rare diseases, the approval speed of orphan drugs, and the coverage of orphan drug improved rapidly after the implementation of a catalog-based management system (Kang et al., 2019; Qiao et al., 2022). Our study supports these findings, indicating a consistent annual increase in the number of specific therapeutic drugs, with several drugs experiencing explosive growth in procurement volume.

The availability of a drug on the market does not guarantee its accessibility for use. In Shandong Province, only 80% of the specific drugs listed in China are accessible, and there are currently 8 drugs in short supply. When considering epidemiological data, at least five diseases sufferers a lack of specific drugs available for treatment in the area. Among the diseases for which drugs are available in Shandong Province, the distribution rate of 16 drugs falls below 80%. Further investigation is warranted to identify issues in the production and distribution of these drugs, and concerted efforts are needed to enhance their availability.

The number of drugs has been increasing annually, but the total procurement expenditure has been decreasing. This trend is likely linked to the national medical insurance policy. Since 2015, China has embarked on a process to negotiate the inclusion of drugs in the national health insurance drug list. This process relies on price negotiations and strategic purchasing mechanisms to incorporate drugs into basic medical insurance coverage (Zhao et al., 2023; Tambuyzer et al., 2020; Yi et al., 2023; Tang et al., 2020). Research indicates that negotiated drug prices have been reduced by an average of over 60% (Chen, 2021). By 2022, 29 rare disease drugs had been included in the national medical insurance through such negotiations (Bi et al., 2023). Notable examples include the price reduction of Nusinersen Sodium Injection from 699,700 RMB to 33,180 RMB, Temozolomide Capsules from 19,675.8 RMB to 246.5 RMB, and Agalsidase Beta from 37,800 RMB to 34,000 RMB. Additionally, various regions have introduced supplementary medical insurance to further alleviate patients’ financial burdens. In Shandong Province, drugs for treating Gaucher Disease, Pompe Disease, and Fabry Disease have been included in the major disease medical insurance coverage (Liu M. et al., 2024).

There is a significant disparity in the quantity and procurement amounts of specific drugs to rare diseases across different regions, which may be attributed to the uneven distribution of medical resources (Qin et al., 2024; Fan et al., 2024). Provincial capital cities, usually characterized by robust economic development, significant political status, and abundant medical resources, tend to have a greater availability of rare disease medications. The findings suggest that while encouraging patients with rare diseases to seek treatment in higher-level medical institutions, regional diagnostic and therapeutic cooperation network for rare diseases could effectively optimize the allocation of medical resources.

Our study innovatively compiled a comprehensive catalogue of specific therapeutic drugs for rare diseases through a meticulous analysis of the first and second lists of rare diseases in China. This catalogue serves as a fundamental resource, enabling medical staff to rapidly grasp the corresponding therapeutic drugs for rare diseases. The analysis of the purchasing data regarding these drugs fills a significant void in the research on downstream drug supply and utilization. Moreover, it provides direct evidence for the expansion of drug coverage and the decrease in costs under the impact of policies. Additionally, regional disparities in the accessibility of rare disease drugs warrant attention.

However, it should be acknowledged that there are certain limitations. Firstly, the study proposed a definition of specific therapeutic drugs for rare diseases without an authoritative standard. Although two researchers independently conducted searches for the included drugs, the rarity and uncommon nature of rare diseases may have resulted in some drugs being overlooked. Secondly, the study did not take into account the timing of drug market entry, which may lead to some drugs purchase quantity is zero may be because the drug is not yet listed at the time. Additionally, the procurement data utilized does not directly equate to usage data. However, considering that orphan drugs are generally high-priced and low-usage and medical institutions typically do not maintain routine stockpiles, we believe that purchasing data can reflect the drug use to some extent.

In conclusion, this study has compiled a catalogue of specific therapeutic drugs for rare diseases by analyzing the first and second lists of rare disease. The analysis of procurement data in Shandong Province reveals a rapid increase in the variety of drugs procured in recent years, accompanied by explosive growth in the volume of multiple drug procurements. While 80% of specific therapeutic drugs are available locally, there is a need to further enhance drug supply capacity, particularly for drugs that are in short supply or have low delivery rates. Additionally, significant disparities exist in drug purchase amounts across different prefecture-level cities.

## Data Availability

The original contributions presented in the study are included in the article/Supplementary Material, further inquiries can be directed to the corresponding author.

## References

[B1] Adachi T. El-Hattab A. W. Jain R. Nogales Crespo K. A. Quirland L. C. I. Scarpa M. (2023). Enhancing equitable access to rare disease diagnosis and treatment around the world: a review of evidence, policies, and challenges. Int. J. Environ. Res. Public Health 20 (6), 4732. 10.3390/ijerph20064732 36981643 PMC10049067

[B2] Barrett J. S. Betourne A. Walls R. L. Lasater K. Russell S. Borens A. (2023). The future of rare disease drug development: the rare disease cures accelerator data analytics platform (RDCA-DAP). J. Pharmacokinet. Pharmacodyn. 50 (6), 507–519. 10.1007/s10928-023-09859-7 37131052 PMC10673974

[B3] Bi Y. Wu M. Wen Z. Kong L. Gong X. (2023). Overview and analysis of rare disease drugs in the national drug list. World Clin. Drug 44 (10), 1098–1103. 10.13683/j.wph.2023.10.014

[B4] Burgos C. M. Irvine W. Vivanti A. Conner P. Machtejeviene E. Peters N. (2024). European reference network for rare inherited congenital anomalies (ERNICA) evidence based guideline on the management of gastroschisis. Orphanet J. Rare Dis. 19 (1), 60. 10.1186/s13023-024-03062-8 38347519 PMC10860293

[B5] Chen L. (2021). The average price reduction negotiated by medical insurance was 61.71%, and many rare disease drugs entered medical insurance. Secur. Times (A03). 10.38329/n.cnki.nzjsb.2021.005146

[B6] Chen R. Liu S. Han J. Zhou S. Liu Y. Chen X. (2024). Trends in rare disease drug development. Nat. Rev. Drug Discov. 23 (3), 168–169. 10.1038/d41573-023-00177-8 37932437

[B7] Chen S. C. Perfect J. Colombo A. L. Cornely O. A. Groll A. H. Seidel D. (2021). Global guideline for the diagnosis and management of rare yeast infections: an initiative of the ECMM in cooperation with ISHAM and ASM. Lancet Infect. Dis. 21 (12), e375–e386. 10.1016/S1473-3099(21)00203-6 34419208

[B8] Chung C. C. Y. Chu A. T. W. Chung B. H. Y. Hong Kong Genome Project (2022). Rare disease emerging as a global public health priority. Front. Public Health 18, 10. 10.3389/fpubh.2022.1028545 PMC9632971 36339196

[B9] de Graeff N. Groot N. Brogan P. Ozen S. Avcin T. Bader-Meunier B. (2019). European consensus-based recommendations for the diagnosis and treatment of rare paediatric vasculitides - the SHARE initiative. Rheumatol. Oxf. 58 (4), 656–671. 10.1093/rheumatology/key322 30535249

[B10] Fan Z. Yang P. Zheng C. Wang T. Yin J. Sun Q. (2024). Evaluation on the health service resources allocation efficiency in Shandong province under hierarchical diagnosis and treatment. Chin. Health Econom. 43 (04), 42–46.

[B11] Feng J. Liu L. Li S. An J. (2021). Medical security for rare diseases in Japan and its enlightenment to China. China J. Pharm. Econ. 16 (01), 27–30. 10.12010/j.issn.1673-5846.2021.01.005

[B12] Feng J. N. Guo J. Z. Zhang Q. Zhuo L. Xu L. Liu L. L. (2023). Higher prevalence of generalized pustular Psoriasis in asia? A population-based study using claim data in China and a systematic review. Dermatology 239 (2), 195–205. 10.1159/000528850 36592625

[B13] Giannuzzi V. Conte R. Landi A. Ottomano S. A. Bonifazi D. Baiardi P. (2017). Orphan medicinal products in Europe and United States to cover needs of patients with rare diseases: an increased common effort is to be foreseen. Orphanet J. Rare Dis. 12 (1), 64. 10.1186/s13023-017-0617-1 28372595 PMC5376695

[B14] Gorini F. Santoro M. Pierini A. Mezzasalma L. Baldacci S. Bargagli E. (2022). Orphan drug use in patients with rare diseases: a population-based cohort study. Front. Pharmacol. 13, 869842. 10.3389/fphar.2022.869842 35652051 PMC9148958

[B15] Haendel M. Vasilevsky N. Unni D. Bologa C. Harris N. Rehm H. (2020). How many rare diseases are there? Nat. Rev. Drug Discov. 19 (2), 77–78. 10.1038/d41573-019-00180-y 32020066 PMC7771654

[B16] He J. Kang Q. Hu J. Song P. Jin C. (2018). China has officially released its first national list of rare diseases. Intractable Rare Dis. Res. 7 (2), 145–147. 10.5582/irdr.2018.01056 29862160 PMC5982625

[B17] Heard J. M. Vrinten C. Schlander M. Bellettato C. M. van Lingen C. Scarpa M. MetabERN collaboration group (2020). Availability, accessibility and delivery to patients of the 28 orphan medicines approved by the European Medicine Agency for hereditary metabolic diseases in the MetabERN network. Orphanet J. Rare Dis. 15 (1), 3. 10.1186/s13023-019-1280-5 31907071 PMC6945588

[B18] Kang Q. Hu J. Yang N. He J. Yang Y. Tang M. (2019). Marketing of drugs for rare diseases is speeding up in China: looking at the example of drugs for mucopolysaccharidosis. Intractable Rare Dis. Res. 8 (3), 165–171. 10.5582/irdr.2019.01090 31523593 PMC6743428

[B19] Liu M. Lu Y. Li J. Zhang Y. Zhang S. Liu Y. (2024a). Orphan drug policy analysis in China. Front. Pharmacol. 15, 1278710. 10.3389/fphar.2024.1278710 38939834 PMC11208459

[B20] Liu Q. Liu X. Wang S. Shang J. Tang Y. Zhang Bo (2023). Research of accessibility of rare disease drugs based on the China’s first list of rare diseases. Med. J. Peking Union Med. Coll. Hosp. 14 (06), 1208–1216. 10.12290/xhyxzz.2023-0163

[B21] Liu Q. Liu X. Zuo W. Wang S. Zhang Bo Zhang S. (2024b). Study on drug list and accessibility of rare diseases based on the China’s second list of rare diseases. J. Rare Dis. 3 (02), 195–201. 10.12376/j.issn.2097-0501.2024.02.007

[B22] Lu Y. Han J. (2022). The definition of rare disease in China and its prospects. Intractable Rare Dis. Res. 11 (1), 29–30. 10.5582/irdr.2022.01034 35261848 PMC8898396

[B23] Nguengang W. S. Lambert D. M. Olry A. Rodwell C. Gueydan C. Lanneau V. (2020). Estimating cumulative point prevalence of rare diseases: analysis of the Orphanet database. Eur. J. Hum. Genet. 28 (2), 165–173. 10.1038/s41431-019-0508-0 31527858 PMC6974615

[B24] Puig-Junoy J. Campillo-Artero C. (2019). Orphan drugs. Lancet 393 (10181), 1595. 10.1016/S0140-6736(19)30017-0 31007200

[B25] Qiao L. Liu X. Shang J. Zuo W. Xu T. Qu J. (2022). Evaluating the national system for rare diseases in China from the point of drug access: progress and challenges. Orphanet J. Rare Dis. 17 (1), 352. 10.1186/s13023-022-02507-2 36088349 PMC9463840

[B26] Qin A. Qin W. Hu F. Wang M. Yang H. Li L. (2024). Does unequal economic development contribute to the inequitable distribution of healthcare resources? Evidence from China spanning 2001-2020. Glob. Health 20 (1), 20. 10.1186/s12992-024-01025-z PMC10913684 38443966

[B27] Rare diseases (2022). Common challenges. Nat. Genet. 54 (3), 215. 10.1038/s41588-022-01037-8 35288710

[B28] Richter T. Nestler-Parr S. Babela R. Khan Z. M. Tesoro T. Molsen E. International Society for Pharmacoeconomics and Outcomes Research Rare Disease Special Interest Group (2015). Rare disease terminology and definitions-A systematic global review: report of the ISPOR rare disease special interest group. Value Health 18 (6), 906–914. 10.1016/j.jval.2015.05.008 26409619

[B29] Tambuyzer E. Vandendriessche B. Austin C. P. Brooks P. J. Larsson K. Miller Needleman K. I. (2020). Therapies for rare diseases: therapeutic modalities, progress and challenges ahead. Nat. Rev. Drug Discov. 19 (2), 93–111. 10.1038/s41573-019-0049-9 31836861

[B30] Tang M. Song P. He J. (2020). Progress on drug pricing negotiations in China. Biosci. Trends 13 (6), 464–468. 10.5582/bst.2019.01339 31875587

[B31] Tang M. Yang Y. Ye Z. Song P. Jin C. Kang Q. (2023). Release and impact of China’s second list of rare diseases. Intractable Rare Dis. Res. 12 (4), 251–256. 10.5582/irdr.2023.01086 38024584 PMC10680157

[B32] The Lancet (2020). Rare diseases need sustainable options. Lancet. 29 (10225), 395. 10.1016/S0140-6736(20)30457-8 32113490

[B33] The Lancet Neurology (2022). Rare diseases: maintaining momentum. Lancet Neurol. 21 (3), 203. 10.1016/S1474-4422(22)00046-1 35182497

[B34] Yang Y. Kang Q. Hu J. Kong F. Tang M. He J. (2019). Accessibility of drugs for rare diseases in China: policies and current situation. Intractable Rare Dis. Res. 8 (2), 80–88. 10.5582/irdr.2019.01068 31218157 PMC6557233

[B35] Yang Y. Xie J. Shao R. (2022). Introduction of the prevention and guarantee system of rare diseases in South Korea and its enlightenment to China. China Pharm. 33 (22), 2689–2693. 10.6039/j.issn.1001-0408.2022.22.01

[B36] Yi H. Shi F. Wang Z. Kuai L. Xu D. Xie Y. (2023). Impacts of adjustment of National Reimbursement Drug List on orphan drugs volume and spending in China: an interrupted time series analysis. BMJ Open 13 (10), e064811. 10.1136/bmjopen-2022-064811 PMC10603398 37852769

[B37] Zhang S. Chen L. Zhang Z. Zhao Y. (2019). Orphan drug development in China: progress and challenges. Lancet 394 (10204), 1127–1128. 10.1016/S0140-6736(19)32179-8 31571591

[B38] Zhao Z. Pei Z. Hu A. Zhang Y. Chen J. (2023). Analysis of incentive policies and initiatives on orphan drug development in China: challenges, reforms and implications. Orphanet J. Rare Dis. 18 (1), 220. 10.1186/s13023-023-02684-8 37501126 PMC10375655

[B39] Zhi W. Liu M. Yang D. Zhang S. Lu Y. Han J. (2023). Analysis of marketed orphan drugs in China. Intractable Rare Dis. Res. 12 (3), 132–140. 10.5582/irdr.2023.01030 37662620 PMC10468412

