# Supplementary material for: Specific drugs for rare diseases in a province of eastern China under catalog management: from 2021 to 2023
Source: Front Pharmacol. 2025 Mar 31;16:1476910. doi: 10.3389/fphar.2025.1476910 (PMC11994414; doi:10.3389/fphar.2025.1476910)
Supplement: Supplementary file 1 [file Table1.docx]

**Specific drugs for rare diseases in a province of eastern China under catalog management: from 2021 to 2023**

Ruifang Nie^1^, Zhen Zhao^1^, Yahui Zhang^1^, Bo Xu^2^, Wen Zhang^1^*

*^1^Department of Pharmacy,* *Shandong Provincial Hospital Affiliated to Shandong First Medical University, Jinan, 250021, China, ^2^Shandong Provincial Public Resources Trading Center*

*Correspondence: Wen Zhang, E-mail address: zhangwen_jky@126.com

Contents

[1. China’s First List of Rare Diseases and Specific Therapeutic Drugs 2](#_Toc173352894)

[2. China’s Second List of Rare Diseases and Specific Therapeutic Drugs 4](#_Toc173352895)

# 1. China’s First List of Rare Diseases and Specific Therapeutic Drugs

Table **S1** China’s First List of Rare Diseases and specific therapeutic drugs

| **Entry Catalog** | **Rare Disease** | **Drug** | **Allocation** |
| --- | --- | --- | --- |
| 4 | Amyotrophic Lateral Sclerosis | Riluzole | √ |
| 8 | Atypical Hemolytic Uremic Syndrome | Eculizumab | √ |
| 16 | Castleman Disease | Siltuximab | √ |
| 27 | Fabry Disease | Agalsidase Alfa | √ |
|  |  | Agalsidase Beta | √ |
| 31 | Gaucher’s Disease | Imiglucerase | √ |
| 32 | Generalized Myasthenia Gravis | Eculizumab | √ |
| 35 | Glycogen Storage Disease (Type I、II） | Alglucosidase Alfa | √ |
| 36 | Hemophilia | Eftrenonacog Alfa | × |
|  |  | Recombinant Coagulation Factor IX | × |
|  |  | Recombinant Human Coagulation Factor VIIa | √ |
|  |  | Emicizumab | √ |
| 38 | Hereditary Angioedema (HAE) | lcatibant | √ |
|  |  | Lanadelumab | × |
|  |  | Danazol | √ |
| 46 | Homozygous Hypercholesterolemia | Alirocumab | √ |
|  |  | Evolocumab | √ |
| 47 | Huntington Disease | Deutetrabenazine | √ |
|  |  | Tetrabenazine | × |
| 49 | Hyperphenylalaninemia | Sapropterin | √ |
| 51 | Hypophosphatemic Rickets | Burosumab | √ |
| 52 | Idiopathic Cardiomyopathy | Tafamidis | √ |
| 54 | Idiopathic Pulmonary Arterial Hypertension | Ambrisentan | √ |
|  |  | Bosentan | √ |
|  |  | Selexipag | √ |
|  |  | Macitentan | √ |
|  |  | Treprosti | √ |
|  |  | Inhalation | × |
|  |  | Riociguat | √ |
| 55 | Idiopathic Pulmonary Fibrosis | Nintedanib | √ |
|  |  | Pirfenidone | √ |
| 64 | Lymphangioleiomyomatosis (LAM) | Sirolimus | √ |
| 73 | Mucopolysaccharidosis | Laronidase | √ |
|  |  | Elosulfase Alfa | × |
|  |  | Idursulfase Beta | √ |
| 76 | Multiple Sclerosis | Siponimod | √ |
|  |  | Fingolimod | √ |
|  |  | Fampridine | √ |
|  |  | Teriflunomide | √ |
|  |  | Baclofen | √ |
|  |  | Dimethyl Fumarate | √ |
|  |  | Ofatumumab | √ |
|  |  | Glatiramer | × |
|  |  | Fampridine | × |
|  |  | Ozanimod | × |
| 79 | N-acetylglutamate Synthase Deficiency | Sodium Phenylbutyrate | × |
| 81 | Neuromyelitis Optica | Inebilizumab | √ |
|  |  | Eculizumab | √ |
| 82 | Niemann-Pick Disease | Miglustat | √ |
| 85 | Ornithine Transcarbamylase Deficiency | Sodium Phenylbutyrate | × |
| 86 | Osteogenesis Imperfecta (Brittle Bone Disease) | Denosumab | √ |
| 88 | Paroxysmal Nocturnal Hemoglobinuria | Eculizumab | √ |
| 90 | Phenylketonuria | Sapropterin | √ |
| 96 | Primary Light Chain Amyloidosis | Melphalan | √ |
| 105 | Severe Myoclonic Epilepsy in Infancy (Dravet Syndrome) | Clobazam | √ |
|  |  | Stiripentol | × |
| 110 | Spinal Muscular Atrophy | Nusinersen Sodium | √ |
|  |  | Risdiplam | √ |
| 112 | Systemic Sclerosis | Nintedanib | √ |
| 113 | Tetrahydrobiopterin Deficiency | Sapropterin | √ |
| 114 | Tuberous Sclerosis Complex | Everolimus | √ |
|  |  | Sirolimus | √ |
| 115 | Tyrosinemia | Nitisinone | × |

# 2. China’s Second List of Rare Diseases and Specific Therapeutic Drugs

Table **S2** China’s Second List of Rare Diseases and specific therapeutic drugs

| **Entry Catalog** | **Rare Disease** | **Drug** | **Allocation** |
| --- | --- | --- | --- |
| 2 | Acquired Hemophilia | Recombinant Human Coagulation Factor VIIa | √ |
| 3 | Acromegaly | Octreotide | √ |
|  |  | Lanreotide | √ |
| 7 | ANCA-associated Vasculitis | Mepolizumab | √ |
| 20 | Cutaneous T-cell Lymphomas | Brentuximab Vedotin | √ |
|  |  | Mogamulizumab | × |
| 26 | Familial Hemophagocytic Lymphohistiocytosis | Emapalumab | × |
| 31 | Gastroenteropancreatic Neuroendocrine Neoplasm | Octreotide | √ |
|  |  | Sunitinib | √ |
| 32 | Gastrointestinal Stromal Tumor | Ripretinib | √ |
| 33 | Generalized Pustular Psoriasis | spesolimab | × |
| 36 | Giant Cell Tumor of Bone | Denosumab | √ |
|  |  | Narlumosbart | √ |
| 38 | Glioblastoma | Temozolomide | √ |
| 44 | Lennox-Gastaut Syndrome | Clobazam | √ |
| 46 | Malignant Hyperthermia | Dantrolene Sodium | × |
| 48 | Melanoma | Ipilimumab | √ |
|  |  | Pembrolizumab | √ |
|  |  | Dabrafenib | √ |
|  |  | Trametinib | √ |
|  |  | Vemurafenib | √ |
| 52 | Narcolepsy | Pitolisant | × |
| 53 | Neuroblastoma | Naxitamab | √ |
|  |  | Dinutuximab Beta | √ |
| 54 | Neurofibromatosis | Selumetinib | √ |
| 56 | Neurotrophic Keratitis | Cenegermin | × |
| 59 | Persistent Pulmonary Hypertension of the Newborn | Nitric Oxide | × |
| 64 | Primary Growth Hormone Deficiency | Recombinant Human Growth Hormone | √ |
|  |  | Polyethylene Glycol Recombinant Human Somatropin | √ |
| 67 | Primary Myelofibrosis | Ruxolitinib | √ |
| 69 | Progressive Fibrosing Interstitial Lung Disease | Nintedanib | √ |
| 71 | Retinopathy of Prematurity | Ranibizumab | √ |
| 78 | Thalassemia Major | Luspatercept | √ |
| 80 | Transthyretin Amyloidosis | Tafamidis | √ |
| 82 | Tumor-induced Osteomalacia | Burosumab | √ |
| 86 | West Syndrome/Infantile Spasms Syndrome | Vigabatrin | √ |
